# Perceived occurrence of an adverse event affects patient-reported outcomes after total hip replacement

**DOI:** 10.1186/s12891-020-3127-6

**Published:** 2020-02-21

**Authors:** Charlotte V. E. Carpenter, Vikki Wylde, Andrew J. Moore, Adrian Sayers, Ashley W. Blom, Michael R. Whitehouse

**Affiliations:** 1 Musculoskeletal Research Unit, Translational Health Sciences, Bristol Medical School, 1st Floor Learning & Research Building, Southmead Hospital, Bristol, BS10 5NB UK; 2 National Institute for Health Research Bristol Biomedical Research Centre, University Hospitals Bristol NHS Foundation Trust and University of Bristol, Bristol, UK

**Keywords:** Total hip replacement, Outcomes, Adverse events, Patient reporting

## Abstract

**Background:**

Dislocation, periprosthetic fracture and infection are serious complications of total hip replacement (THR) and which negatively impact on patients’ outcomes including satisfaction, quality of life, mental health and function. The accuracy with which patients report adverse events (AEs) after surgery varies. The impact of patient self-reporting of AEs on patient-reported outcome measures (PROMs) after THR is yet to be investigated. Our aim was to determine the effect of confirmed and perceived AEs on PROMs after primary THR.

**Methods:**

A prospective single-centre cohort study of patients undergoing primary THR, with one-year follow-up, was performed. Participants completed forms pre-operatively and 3, 6, 9 and 12 months post-operatively, including Work Productivity and Activity Impairment (WPAI), Western Ontario and McMaster Universities Osteoarthritis Index (WOMAC), EuroQol-5D-3 L (EQ5D), Self-Administered Patient Satisfaction (SAPS) and AE reporting questionnaires. Results were reported in three groups: No AE, reported but not confirmed AE and confirmed AE. A generalised linear model was used to compare among groups using robust standard errors (SE).

**Results:**

Forty-one AEs were reported in a cohort of 417 patients (234 females), with 30 AEs reported by 3 months. Eleven (27 reported) infections, two (six reported) periprosthetic fractures and two (eight reported) dislocations were confirmed. Those in the no AE group reported significantly better outcomes that the reported AE group as measured by WOMAC Co-Eff 14.27 (*p* = 0.01), EQ5D − 0.128 (*p* = 0.02) and SAPS − 9.926 (*p* = 0.036) and the combined reported and confirmed AE groups as measured by WOMAC Co-Eff 13.72 (*p* = 0.002), EQ5D − 0.129 (*p* = 0.036) and SAPS − 11.512 (*p* = 0.004). No significant differences were seen in WPAI among groups.

**Conclusions:**

Patients who report AEs have worse outcomes than those who do not, regardless of whether the AEs can be confirmed by standard medical record review methods. The observed negative trends suggest that patient perception of AEs may influence patient outcome in a similar way to those with confirmed AEs.

## Introduction

In England and Wales approximately 97,000 primary total hip replacements (THR) were performed during 2017 [1]. Demand for THR is likely to increase with an ageing population [2, 3]. THR is a successful option for the treatment of chronic hip pain with 90% of patients satisfied with their outcome [4]. Dislocation, periprosthetic fracture and infection are relatively rare but serious complications of THR. They often require hospital admission, further major operations and ongoing hospital-based care posing a significant burden both to the patient and health care system [5–7].

Adverse events (AEs) significantly impact on patients’ outcomes after THR. Dislocation, particularly recurrent dislocations, can negatively impact on patient satisfaction, quality of life, mental health and function, including self-care and daily activities [8]. After post-operative periprosthetic fracture, approximately 50% of patients do not return to previous levels of mobility and half require assistance with daily living [9]. Prosthetic joint infection often requires major revision surgery and patients experience deeply negative changes in their quality of life enduring severe pain, long periods of immobility, an inability to participate in daily work and leisure activities, social isolation and psychological suffering [10, 11]. In one study, patients with prosthetic joint infection reported poorer outcomes on the Western Ontario and McMaster Universities Osteoarthritis Index (WOMAC), Assessment of Quality of Life (AQoL) and the 36-Item Short Form (SF-36), and 12% of patients rating their current situation equivalent to, or worse than, death [12].

AEs reported by patients may provide information about subjective experiences following a surgical procedure but often reveal different results from AEs recorded in medical records. The accuracy with which patients report AEs after surgical procedures varies widely in the literature. Concordance between patient reports and medical records is between 0 and 58% at 30 days after all surgical procedures [13] and 36–95% at 3 years after hip and knee replacement [14]. Agreement between patient reports and insurance claims after orthopaedic procedures showed poor to moderate agreement (kappa 0 to 0.53) for complications, [15] rising to 69% agreement when patients are telephoned to confirm AEs reported via mail surveys [16].

Previous studies have evaluated the accuracy with which patients report AEs after joint replacement surgery and the impact of the most common AEs on patient-reported outcome measures (PROMs) [13–16]. However, the impact of self-reporting of an AE on PROMs after THR is yet to be investigated.

The aim of this study was to evaluate the effect of perceived and confirmed adverse events on patient-reported outcome measures over the first 12 months after primary THR.

## Patients and methods

**Fig. 1:**
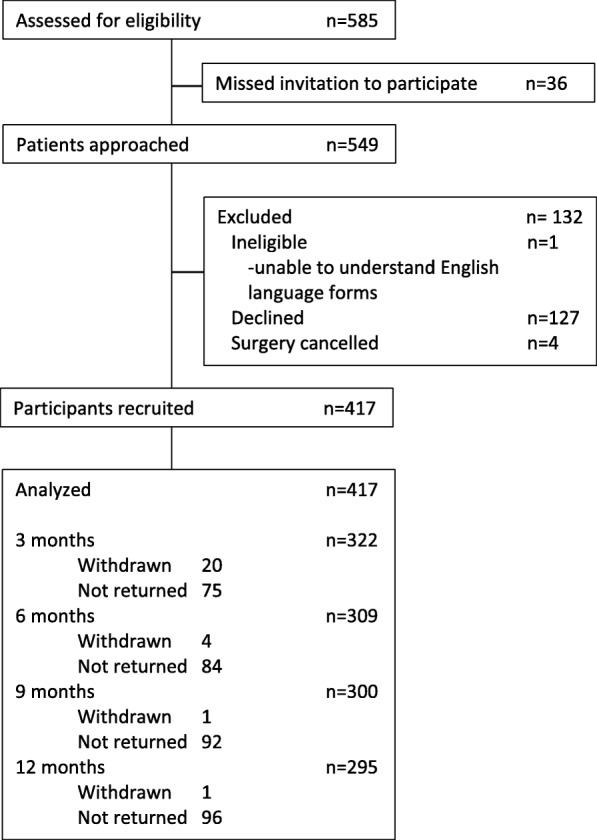
Study flow diagram

A prospective single-centre cohort study was conducted examining the impact of patient reporting of AEs on PROMs following primary THR. Consecutive patients undergoing a primary THR between January 2012 and January 2013 were screened for eligibility prior to attending a preoperative assessment outpatient appointment. Patients meeting the inclusion criteria were invited to participate at this appointment. Patients were eligible for inclusion if they were due to undergo primary elective THR, were able to provide consent to participate and were able to understand and complete the English language questionnaires. Exclusion criteria were patients undergoing revision arthroplasty, patients who were unwilling or unable to provide consent and patients who were unable to understand or complete the questionnaires [Fig. 1]. Participation was voluntary, and patients provided verbal consent to participate. This study was part of a larger service evaluation project of THR and total knee replacement in this centre and ethical approval was not required according to the National Research Ethics Service guidelines. Participants were asked to complete questionnaires at five timepoints: pre-operatively and at 3, 6, 9 and 12 months post-operatively. Clinical follow-up and post-operative rehabilitation were determined by the treating surgeon and not affected by inclusion in this study. The pre-operative questionnaire assessed work status using the Work Productivity and Activity Impairment (WPAI), [17] hip function using the WOMAC [18] and health-related quality of life using the EuroQol-5D-3 L (EQ5D) [19]. The post-operative questionnaire included all the pre-operative questionnaires as well as the Self-Administered Patient Satisfaction scale (SAPS) [20] and an AE reporting questionnaire. The pre-operative questionnaire was administered and completed at a pre-operative assessment outpatient appointment, no more than 60 days prior to the date of the primary THR. Post-operative questionnaires were posted to participants, and if no response was received in 2 weeks, a reminder was sent.

The WPAI is an instrument to measure impairment in both paid and unpaid work. It can be adapted to measure absence and impairment due to a specific health problem [17]. A score displayed as a percentage may be calculated for work time missed, impairment while working, overall work impairment and activity impairment due to the health problem. The WPAI was completed by participants in employment. The percentage activity impairment represents the degree to which the hip due to be or that had been replaced affected regular activities.

WOMAC is a 24-item questionnaire designed to measure pain, function and stiffness in patients with osteoarthritis of the hip or knee [18]. It uses a 5-point Likert scale from 0 to 4 for each question (giving a total scale of 0–96) with higher scores indicating worse outcomes. A percentage score for each subscale of the WOMAC and a total was calculated, giving a score out of 100 for stiffness, pain, function and total WOMAC score.

EQ5D is a standardised, non-disease specific questionnaire for evaluating health-related quality of life in five dimensions, including mobility, self-care, usual activities, pain or discomfort and anxiety or depression [19]. The EQ5D index is derived from a standardised value set to provide a single value for health status, where one represents full health.

SAPS is a short questionnaire used to evaluate patient satisfaction with total hip and knee replacement [20]. Four items are scored on a 4-point Likert scale with responses from very dissatisfied to very satisfied. The scale score is the unweighted mean of the scores with 100 being most satisfied and 25 the least.

Study-specific questionnaires were used to evaluate AEs. Participants were asked if they had any problem with infection in the joint or wound, a dislocation of the THR or a fracture around the THR, after their surgery. Participants were asked to record details of these events and were asked if any other complications had occurred [Appendix 1]. The first report of an AE episode was counted, in the case of infection, if there was an infection that persisted and hence reported at multiple time points, it was only counted once.

For AEs reported by participants hospital systems and primary care records were interrogated to see if the AE could be confirmed. Hospital medical records were searched for hospital admissions and discharge documentation, outpatient clinic letters, operation records, radiology and microbiology reports. Local picture archiving and communications imaging systems were searched for evidence of dislocation or periprosthetic fracture. Primary care records were searched for documentation of an AE, post-operative general practice attendance or antibiotic prescribing at the time of the recorded AE.

Missing data were handled according to the user guide for each PROM. Data were analysed using STATA (version 13, StataCorp, College Station, TX, USA). Results are reported in three groups of participants:

1) No AE group: participants who reported no AE.

2) Reported group: participants who reported an AE which was not confirmed after searching medical records as described above.

3) Confirmed AE group: participants who reported an AE that was confirmed after medical record search.

Data were checked for normal distribution using a Shapiro-Wilks test and histogram plots. Where data were not normally distributed, central tendency is described as median value with inter-quartile range (IQR). PROMs data were compared among groups at 12 months using a generalised linear model(GLR) with robust standard errors (SE) in order to account for the non-normal distribution of data. The models were adjusted to account for age, gender and body mass index (BMI). The first model describes the relationship of the no AE group to the reported group and the confirmed group. The second model was constrained to assume no difference between the reported and confirmed groups and the Akaike information criteria (AIC) between the models were then compared using a likelihood ratio test (LRtest). A *p*-value of < 0.05 was considered statistically significant.

## Results

Pre-operatively 549 patients were invited to participate and 417 were recruited. At first follow-up 322 responses were received. By 12 months, there were a further 27 non-responders and six participants withdrew from the study.

Baseline demographics and PROMs scores are displayed in Table 1 and were similar among the groups.

**Table 1:** Patient demographics and baseline data. (IQR: interquartile range, BMI: body mass index, WPAI: Work Productivity and Activity Impairment, EQ5D: EuroQol-5D-3 L, WOMAC: Western Ontario and McMaster Universities Osteoarthritis Index, SAPS: Self-Administered Patient Satisfaction scale)

Variable	No Adverse Event (*n* = 382)	Reported (*n* = 20)	Confirmed (*n* = 15)	All participants (*n* = 417)
Age (IQR)	68 (57, 75)	61 (57, 75)	67 (50, 75)	67 (57, 75)
Female sex (%)	213 (56%)	12 (60%)	8 (53%)	234 (56%)
BMI (IQR)	28 (25, 31)	33 (29, 36)	30 (26, 35)	28 (25, 32)
Retired (%)	222 (60%)	17 (55%)	7 (50%)	240 (59%)
WPAI (IQR)	70 (50, 80)	70 (70, 90)	65 (40, 80)	70 (50, 80)
EQ5D (IQR)	0.52 (0.06, 0.70)	0.52 (0.6, 0.62)	0.16 (0.02, 0.69)	0.52 (0.6, 0.69)
WOMAC (IQR)	59.9 (50, 69.8)	60.1 (54.2, 77.1)	59.4 (55.2, 70.8)	59 (50, 70)

Forty-one AEs were reported by 35 participants with three participants reporting two AEs and one participant reporting four (Table 2).

**Table 2:** Adverse events by group, reported but not confirmed or confirmed adverse event and timepoint reported. Note: the number of AE (41) is greater than the number of participants that reported AE (35) as some reported more than one event

	INFECTION		PERIPROSTHETIC FRACTURE		DISLOCATION		TOTAL	
ADVERSE EVENTS	Reported	Confirmed	Reported	Confirmed	Reported	Confirmed	Reported	Confirmed
3 MONTHS	13	11	2	1	3	0	**18**	**12**
6 MONTHS	1	0	1	0	1	1	**3**	**1**
9 MONTHS	2	0	0	1	0	1	**2**	**2**
12 MONTHS	0	0	1	0	2	0	**3**	**0**
TOTAL	16	11	4	2	6	2	**26**	**15**

Most (*n* = 30) AEs were reported by 3 months post-operation. Fifteen AEs were confirmed by a review of medical records and 26 could not be confirmed. Eleven AEs were identified on review of secondary care records and a further four, all superficial wound infections treated by the GP, were identified after review of primary care records. Infection was the most commonly reported AE, with the majority (24 of 27) reported at 3 months. Eleven infections were confirmed, 10 were superficial wound infections and one a prosthetic joint infection.

Pre-operatively, PROMs were similar across each of the three groups, except for the EQ5D which was lower in the confirmed AE group. All outcomes in all groups improved with time (Table 3).

**Table 3:** A comparison of patient reported outcomes over time by group. (AE: adverse event, IQR: interquartile range, WPAI: Work Productivity and Activity Impairment, EQ5D: EuroQol-5D-3 L, WOMAC: Western Ontario and McMaster Universities Osteoarthritis Index, SAPS: Self-Administered Patient Satisfaction scale)

	Pre-op Median (IQR)			3 Months Median (IQR)		6 Months Median (IQR)		9 Months Median (IQR)		12 Months Median (IQR)	
			n		n		n		n		n
WPAI											
No AE	70 (50,80)		106	20 (10,30)	76	10 (0,20)	71	0 (0,20)	68	0 (0,20)	72
Reported	70 (70,90)		6	25 (10,30)	6	0 (0,10)	3	0 (0,50)	3	0 (0,20)	3
Confirmed	65 (40,80)		4	30 (20,40)	4	20 (0,20)	3	20 5,30)	4	15 (5,25)	4
EQ5D											
No AE	0.52 (0.6, 0.69)		395	0.76 (0.62, 1)	313	0.8 (0.69, 1)	298	0.81 (0.69,	285	0.88 (0.71, 1)	289
Reported	0.52 (0.16, 0.59)		18	0.62 (0.52, 0.76)	17	0.7 (0.55, 0.91)	16	0.7 (0.62, 0.78)	14	0.7 (0.52, 0.82)	14
Confirmed	0.16 (0.02, 0.69)		15	0.59 (0.36,0.76)	15	0.75 (0.64, 1)	12	0.7 (0.22, 0.91)	12	0.76 (0.71, 1)	13
WOMAC											
No AE	59.9 (50, 69.8)		364	16.7 (8.3, 31.2)	269	12.5 (4.4, 27.1)	267	10.4 (3.1, 23)	256	7.3 (2.1, 16.8)	256
Reported	60.1 (54.2, 77.1)		18	29.2 (15.2, 40.1)	16	22.9 (8.3, 50)	17	26 (12.5, 40.1)	16	22.4 (7.3, 46.9)	14
Confirmed	59.4 (55.2, 70.8)		15	25.9 (15.6, 51)	15	21.7 5.2, 33.3)	11	26.5 (6.5, 29.9)	12	15.6 (5.2, 29.1)	13
SAPS											
No AE				93.8 (81.3, 100)	268	100 (81.3, 100)	250	100 (81.3, 100)	247	100 (87.5, 100)	248
Reported				78.1 (75, 100)	18	87.5 (68.8, 100)	17	93.8 (75, 100)	15	87.5 (75, 100)	14
Confirmed				81.3 (68.8, 100)	13	81.3 (71.9, 100)	12	87.5 (62.5, 100)	12	81.3 (62.5, 100)	11

**Fig. 2:**
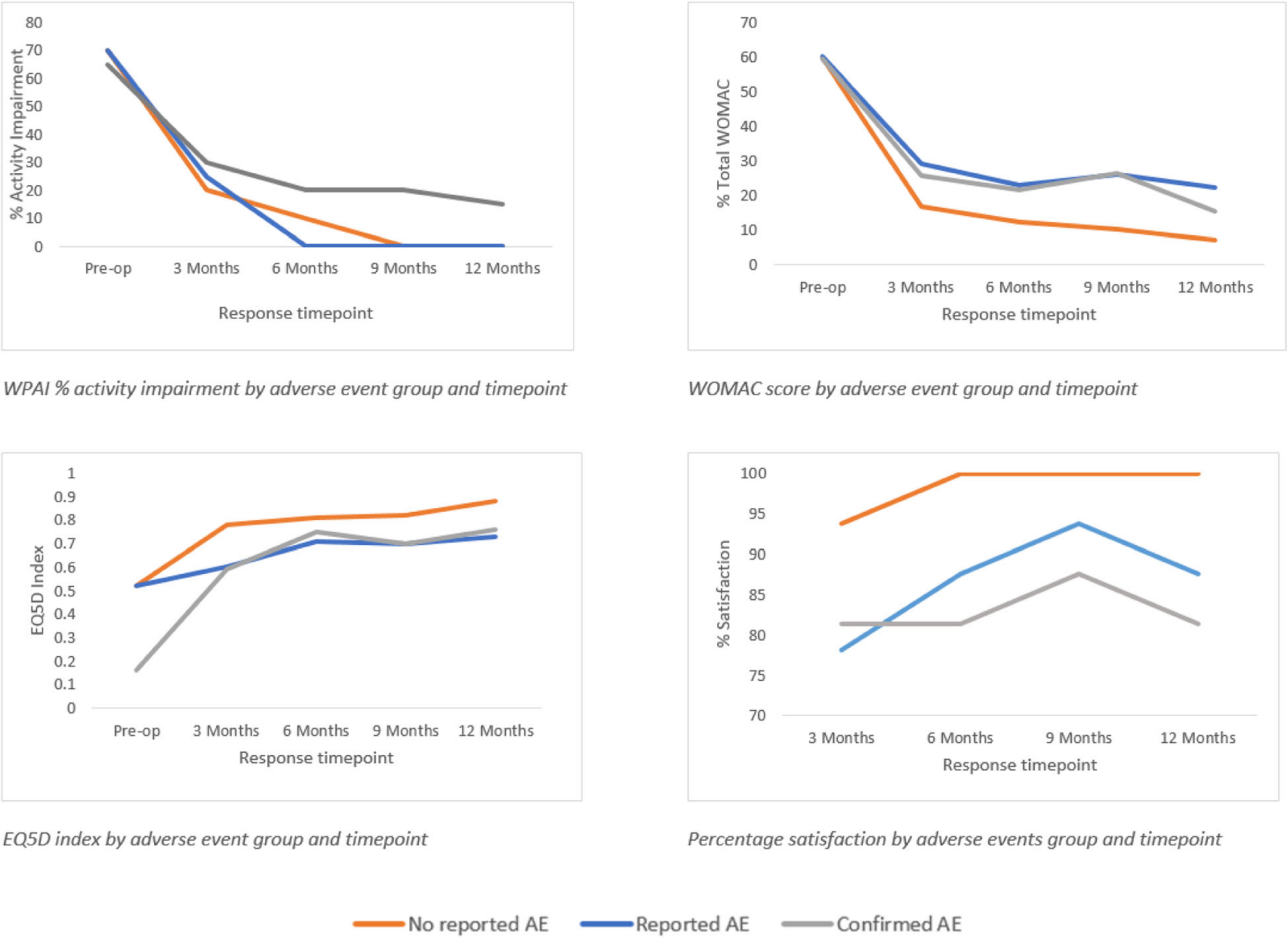
PROMs by adverse event group and timepoint. (WPAI: Work Productivity and Activity Impairment; WOMAC: Western Ontario and McMaster Universities Osteoarthritis Index; EQ5D:EuroQol-5D-3 L)

All PROMs showed the greatest improvement in the first 3 months post-operatively. At each post-operative time point, the confirmed AE and reported AE group showed less improvement than the no AE group for WOMAC, EQ5D and SAPS (Fig. 2). By 6 months the no AE and reported AE groups had no activity impairment associated with their THR.

PROMs at the end-point of 12 months were compared among groups (Table 4).

**Table 4:** A comparison of patient reported outcome measures among groups at 12-months; Model 1: GLR describing the relationship of the no AE group to the reported group and the confirmed group. Model 2: GLR describing the relationship of the no AE group to the reported and confirmed group, when constrained to assume no difference between these two groups. Group 1: No AE group, Group 2: Reported group, Group 3: Confirmed group. (WOMAC: Western Ontario and McMaster Universities Osteoarthritis Index, EQ5D: EuroQol-5D-3 L, SAPS: Self-Administered Patient Satisfaction scale, WPAI: Work Productivity and Activity Impairment)

PROM	Model	Groups	Co-efficient	95%CI	*p*-value	AIC	LRtest
WOMAC	Model 1:	1 to 2	14.27	(3.2, 25.3)	*p* = 0.01	8.5	
		1 to 3	13.13	(−0.68, 26.9)	*p* = 0.06		
	Model 2:	1 to (2 and 3)	13.72	(4.8, 22.6)	*p* = 0.002	8.49	0.85
EQ5D	Model 1:	1 to 2	−0.128	(− 0.25, − 0.006)	*p* = 0.04	0.015	
		1 to 3	−0.129	(−0.34, 0.079)	*p* = 0.22		
	Model 2:	1 to (2 and 3)	−0.1285	(0.25, −0.008)	*p* = 0.036	0.0081	0.9
SAPS	Model 1:	1 to 2	−9.926	(−19.2, −0.7)	*p* = 0.036	8.11	
		1 to 3	−13.5	(−26.6, −0.5)	*p* = 0.042		
	Model 2:	1 to (2 and 3)	−11.515	(−19.4, −3.7)	*p* = 0.004	8.1	0.5
WPAI	Model 1:	1 to 2	−6.805	(−18.7, 5.2)	*p* = 0.264	9.05	
		1 to 3	1.53	(−1.7, 13.7)	*p* = 0.806		
	Model 2:	1 to (2 and 3)	−2.043	(−11.9, 7.82)	*p* = 0.685	9.03	0.6

Both the WOMAC and EQ5D demonstrated a significantly better score for the no AE group when compared to the reported groups. Both WOMAC and EQ5D demonstrated a significantly improved between the no AE group and the reported and confirmed groups in the constrained model and the AIC and LRtest indicate equivalence between the reported and confirmed groups. The no AE group showed significantly better satisfaction than the reported and confirmed groups and the comparison of models suggests there may be a difference among all three results. WPAI demonstrated no significant differences among groups.

## Discussion

In this study, 41 AEs were reported by 35 patients from a cohort of 417 patients undergoing primary THR. Fifteen of the reported AEs were confirmed by primary and secondary care medical records. Most AEs were reported by 3 months post-operatively. Eleven infections, two periprosthetic fractures and two dislocations were confirmed. Participants in the reported AE group and confirmed AE group had similar PROMs at each timepoint, worse than those who did not report an AE.

Three quarters of reported and two thirds of confirmed AEs were reported by 3 months. Most studies have differing follow-up end points, and early reporting of AEs varies. Blom et al. reported 64% of dislocations occur within the first 3 months with 8–11 year follow-up [6] and Phillips et al. reported 90% of dislocations and 85% of infections had occurred within 3 months, with follow-up of 6 months [21]. Infection represented 11 of 12 confirmed AEs at 3 months, 10 of which were superficial wound infections. Surgical site infection after total hip and knee replacement is diagnosed at a median 17 (11–23) days post-operatively [22] and Lamagni reported that 85% of infections occur within the first 30 days of surgery [23]. In this study, none of the confirmed superficial wound infections were subsequently revised for deep prosthetic infection. Reporting of periprosthetic fractures follows a different pattern, with half of all fractures occurring intra-operatively, 24% of which are identified post-operatively [24]. In our study, fractures identified at the time of operation were excluded, however both confirmed fractures occurred at the time of surgery but were not reported until subsequent follow-up. Post-operative periprosthetic fracture occurs in 1.7% of primary THR with a linear survival curve over 10 years, suggesting that the rate of fracture does not change over time [25]. The timing of the AEs reported in our study are in keeping with the published literature.

Fifteen of a reported 41 AEs were confirmed in our study. There are differences between self-reported AE rates and those confirmed by medical records, but rates vary in the literature. One study confirming patient-reported post-operative complications using medical records found agreement in 0–41% of cases [13]. Surgical site infection had agreement (kappa) of 0.53 (95%CI, 0.17 to 0.89) and 0 for fracture/dislocation in a study of internet-based patient reporting across orthopaedic procedures [15]. Agreement tends to be higher when patients are contacted by telephone to confirm complications which may represent confounding as patients are confirming their own self-reports. However, it may be that patients misreport AE due to lack of comprehension or literacy when asked to complete a questionnaire, rather than by an interviewer. When patients were contacted by telephone by a surgeon, to confirm reported AEs, a concordance of 69% was achieved [16]. Alazzawi et al. confirmed 95% of infections, 52% of dislocations and 57% of periprosthetic fractures after primary hip and knee replacement using medical record review and surveying general practitioners [14]. Our overall agreement of 37% is at the lower end of the spectrum reported to date. Most of the reported AEs in our study were not confirmed on review of medical records. Fritz et al. discovered that participants may accurately report a complication that occurred after surgery which may not have been documented in the medical records [13]. Greenbaum identified that 72% of patients with dislocation and 7% of fractures present to an outside hospital after THR [16]. Despite rigorous exploration of regional medical records, some AEs may not have been confirmed if patients presented to hospitals outside the region, but these should still have been captured by primary care records as hospitals are required to report admissions to hospital to the primary care physician and this, in turn, is documented in the primary care records. However, most unconfirmed AEs are explained by patients who report an AE that did not occur. Reasons for these reports include misinterpretation of symptoms as AEs, erroneously reporting an AE that did not occur or accurately reporting an AE that occurred prior to surgery [13, 16]. A large portion of patients reporting an AE, misreport the occurrence of AEs after THR. Regardless of the reason, self-reporting of an AE appears to be associated with outcome. Studies using medical record review to identify AEs after surgery may, therefore, underestimate the number of patients negatively impacted by their surgical procedure.

In this study, participants who reported an AE had outcomes similar to those with a confirmed AE. Both WOMAC and EQ5D demonstrated equivalence between the reported and confirmed groups. SAPS, WOMAC and EQ5D have been shown to be significantly worse in the reported and confirmed groups compared to those with no AE. Self-reporting an AE may be due to patient perception of an AE that did not occur. This appears to negatively impact on outcome. The nocebo effect, a negative expectation derived from a clinical encounter, can adversely influence quality of life. Clinician disclosure about potential side effects of medications can itself contribute to reporting AEs, but this effect has not been investigated in the surgical setting [26]. The cyclical nature of negative perceptions around health, mental well-being and outcomes are echoed by Perrucio et al. who demonstrated that worse self-reported general health scores predicts less improvement after THR. The patient’s perception of health predicts future physical, mental and social outcomes, and this in turn is predicted by the patient’s mental well-being [27]. The negative trends seen across WOMAC, EQ5D and patient reported satisfaction, in this study, suggest that patient perception of AEs may influence health outcome.

One aspect that was not investigated as part of this study were the effects of depression on the perception of an AE. Patients with depression have worse pre and post-operative pain and functional scores but experience the same benefit from THR than those without [28, 29]. The complex interplay of mental well-being, patient perceptions and the impact of AEs with patients’ outcomes after THR requires further investigation. Understanding patient perception and identification of AEs may be more thoroughly investigated using qualitative methods designed to gain an in-depth understanding of patients’ understanding of AE, experiences, opinions regarding healthcare and the impact of these AEs. An investigation of the impact of depression may further explain the trends we have reported.

The findings in this study are generalisable as the baseline demographics of participants in this study are similar to those reported to in the National Joint Registry for England, Wales and Northern Ireland and the Isle of Man [1]. At 12 months, 23% of questionnaires sent to patients were not returned, despite sending out reminders. Although a similar non-response level to previous studies, this may affect the internal validity of the study and thus result in some selection bias [15, 30]. The number of AEs reported and confirmed within this study was relatively small and thus the results of this study should be interpreted with caution. A larger study sample may improve the statistical certainty with which the results can be interpreted.

## Conclusion

In conclusion, patients who report AEs (8%, *n* = 35) have worse outcomes than those who do not. Self-reporting of an AE appears to have a similarly negative impact on outcomes to those with a confirmed AE. Clear information regarding risks and potential AEs, is required, not only for consent, but to ensure patients can correctly identify AEs should they occur. Patients who perceive that they have an AE may require careful monitoring and support.

## Supplementary information

**Additional file 1.** Appendix 1: Adverse events questionnaire sent to patients

## Data Availability

The datasets used and/or analysed during the current study are available from the corresponding author on reasonable request.

## Abbreviations

AE: Adverse event
PROM: Patient reported outcome measure
THR: Total hip replacement
WOMAC: Western ontario and mcmaster universities osteoarthritis index
WPAI: Work productivity and activity impairment
EQ5D: EuroQol-5D-3 L
SAPS: Self-administered patient satisfaction
