# Supplementary material for: Perceived occurrence of an adverse event affects patient-reported outcomes after total hip replacement
Source: BMC Musculoskelet Disord. 2020 Feb 21;21:118. doi: 10.1186/s12891-020-3127-6 (PMC7035750; doi:10.1186/s12891-020-3127-6)
Supplement: Supplementary file 1 — Additional file 1. Appendix 1: Adverse events questionnaire sent to patients [file 12891_2020_3127_MOESM1_ESM.docx]

# Appendix

Appendix 1: Adverse events questionnaire sent to patients

Did you have any problem with infection in the joint or wound after your surgery?

| **No** | **^0^** |  |
| --- | --- | --- |
| **Yes** | **^1^** |  |

| If **Yes**, did your **wound problem** require: | | No^0^ | Yes^1^ |
| --- | --- | --- | --- |
| a. | Extra antibiotics in hospital? |  |  |
| b. | A delay in leaving hospital? |  |  |
| c. | Extra antibiotics from GP? |  |  |
| d. | Re-admission to hospital? |  |  |
|  |  |  |  |
| e. | Other? |  |  |

Has your hip ever dislocated/come out of joint?

| **No** | **^0^** |  |
| --- | --- | --- |
| **Yes** | **^1^** |  |

| If **Yes**: | | No^0^ | Yes^1^ |
| --- | --- | --- | --- |
| a. | Did this happen after a fall? |  |  |
| b. | Did it require a hospital attendance or admission? |  |  |
| c. | Did you need a further operation? |  |  |

| If your hip has dislocated, please give details of the dates this occurred and the hospital you attended for any treatment: |
| --- |
|  |

Have you had a fracture around your hip replacement?

| **No** | **^0^** |  |
| --- | --- | --- |
| **Yes** | **^1^** |  |

| If **Yes**, please give details of the dates this occurred and the hospital you attended for any treatment: |
| --- |
|  |

|  |
| --- |
